# Nitric Oxide and Photosynthesis Interplay in Plant Interactions with Pathogens

**DOI:** 10.3390/ijms26146964

**Published:** 2025-07-20

**Authors:** Elżbieta Kuźniak, Iwona Ciereszko

**Affiliations:** 1 Department of Plant Physiology and Biochemistry, University of Lodz, 90-237 Lodz, Poland; 2 Department of Biology and Plant Ecology, Faculty of Biology, University of Bialystok, 15-245 Bialystok, Poland

**Keywords:** chloroplasts, immune response, infection, plant–pathogen interaction, reactive nitrogen species, S-nitrosylation, stomata immunity

## Abstract

Nitric oxide and reactive nitrogen species are key signalling molecules with pleiotropic effects in plants. They are crucial elements of the redox regulation of plant stress responses to abiotic and biotic stresses. Nitric oxide is known to enhance photosynthetic efficiency under abiotic stress, and reactive nitrogen species-mediated alterations in photosynthetic metabolism have been shown to confer resistance to abiotic stresses. However, knowledge about the role of reactive nitrogen species in plant immune responses remains limited. In this review, we highlight recent advancements in understanding the role of NO in regulating stomatal movement, which contributes to resistance against pathogens. We will examine the involvement of NO in the regulation of photosynthesis, which provides energy, reducing equivalents and carbon skeletons for defence, as well as the significance of protein S-nitrosylation in relation to immune responses. The role of NO synthesis induced in pathogenic organisms during plant–pathogen interactions, along with S-nitrosylation of pathogen effectors to counteract their pathogenesis-promoting activity, is also reported. We will discuss the progress in understanding the interactions between reactive nitrogen species and photosynthetic metabolism, focusing on enhancing crop plants’ productivity and resistance in challenging environmental conditions.

## 1. Introduction

Nitric oxide (NO) and its reactive intermediates, known as reactive nitrogen species (RNS), along with reactive oxygen species (ROS), collectively form a group of redox-active molecules referred to as reactive oxygen and nitrogen species (RONS), which are recognised as versatile biological messengers in plants. At low concentrations, RONS control major physiological processes, such as germination, growth, development, metabolism, and interactions with beneficial microorganisms: legume–*Rhizobium* symbiosis, arbuscular mycorrhizal fungi interactions with plant roots and *Trichoderma*–plant interactions [1,2,3,4,5,6,7]. They also participate in the acclimation of plants to environmental stresses, mediating the defence response to abiotic and biotic stress stimuli [8,9]. Additionally, RONS have been identified as effective plant priming agents that help mitigate environmental stresses such as salinity and drought by regulating defence mechanisms and improving plant physiological performance [10,11,12]. However, the overproduction of RONS under stressful conditions can lead to oxidative and nitrosative stress, resulting in harmful effects in plants, such as cell damage, retarded growth, and development [13,14].

Biotic stress caused by phytopathogenic bacteria, fungi, and viruses has a wide-ranging and negative impact on plants. It causes metabolic disruption, which reduces plant growth, vigour, and productivity in crops and natural flora [15,16]. Pathogens cause substantial economic losses in crop production, thereby reducing food security. Globally, up to 40% of yields in major crops, including maize, rice, and wheat, are lost annually due to pathogens and pests [17].

Photosynthesis is one of the most important physiological processes, and the entire metabolism, along with plant health, overall performance, and productivity in a changing environment, relies on it. Pathogens generally reduce the plant’s photosynthetic capacity in two main ways. First, pathogens that attack green aerial tissues decrease the photosynthetic area by destroying leaf tissue and defoliation. Second, pathogen-induced chlorosis, which is a common disease symptom, reduces the chlorophyll content per chloroplast [18]. However, the molecular mechanisms linking photosynthesis and resistance to pathogens are still poorly recognised. Understanding the physiological and molecular mechanisms underlying plant–pathogen interactions, including those dependent on NO/RNS signalling, is crucial for designing cultivars resistant to pathogens. Moreover, insights into the role of NO/RNS in photosynthesis pave the way for improving photosynthesis to reduce crop losses under biotic stress [19,20].

Plant defence mechanisms against pathogens are complex, including various molecular, cellular, and structural adaptations [21]. Generally, they can be divided into two sub-categories: constitutive and active. The constitutive defence consists of preformed morphological, structural, and chemical barriers [21,22,23,24]. A plant activates the induced defence after it recognises a pathogen. It is expressed at both local and systemic levels, manifesting symptoms of programmed cell death through the hypersensitive response (HR) and the production of pathogenesis-related (PR) proteins, respectively. Plants employ a two-tier innate immune system to defend against pathogens: pattern-triggered immunity (PTI) and effector-triggered immunity (ETI). PTI is activated by the plant’s recognition of conserved pathogen-associated molecular patterns (PAMPs) by plasma membrane-localised pattern recognition receptors (PRRs). Pathogens can overcome PTI by delivering specific effector proteins inside the host plant cells. ETI is triggered when plants recognise these effectors via intracellular immune receptors, primarily the nucleotide-binding leucine-rich repeat (NLR) protein receptors. ETI responses are typically more robust and specific [25,26,27].

An efficient plant defence against the invading pathogen strongly depends on rapidly generating signalling molecules that directly or indirectly trigger a specific cellular resistance mechanism [28]. The generation of RONS is one of the earliest responses activated in plants after pathogen recognition, reported in many plant–pathogen interactions [13,29,30]. The early burst of ROS and RNS occurs in the apoplast and is recognised as a hallmark of plant response to PAMP. The generation of ROS/RNS in the extracellular space influences their intracellular production in chloroplasts, mitochondria, and peroxisomes, which contributes to ETI-related late defence response [31,32,33,34]. Recent studies have expanded our knowledge on the role of ROS and RNS in plant interactions with microorganisms [1,35]. However, we still need a better understanding of ROS/RNS signalling mechanisms and communication between organelles in plant–pathogen interactions. The way plants decode and integrate ROS and RNS signals, both locally in cells at infection sites and in distant parts of the plant, is crucial for reprogramming gene expression and metabolism, ultimately shaping an effective defence response.

This review provides insights into the pathogen-induced interactions between RONS and photosynthesis, as well as related metabolic processes, which are essential for understanding plant–pathogen interactions. The relationship between ROS, RNS, and the primary metabolism is also discussed in the context of sustaining the productivity of crop plants that face pathogen infection.

## 2. RONS Production and Catabolism During Plant-Pathogen Interactions

The generation of reactive species, comprising ROS and RNS, is a frontline plant response to stress. Depending on their concentration, these reactive species may have either a positive or negative impact on the plant. ROS and RNS, produced in chloroplasts, mitochondria, peroxisomes, and the apoplast, can result in nitro-oxidative stress or act as stress-alleviating signalling molecules [36,37,38,39,40].

The mechanisms of ROS generation and action in plants experiencing biotic stress have been thoroughly investigated for decades. Recent advances in understanding the various modes of ROS signalling and management in plants infected by pathogens have been discussed in several reviews [40,41,42]. It has become clear that activating the defence response to pathogen infection requires a complex interaction between ROS and RNS [43]. However, there are still gaps in identifying NO sources and its fate in plants [44,45].

**Figure 1:**
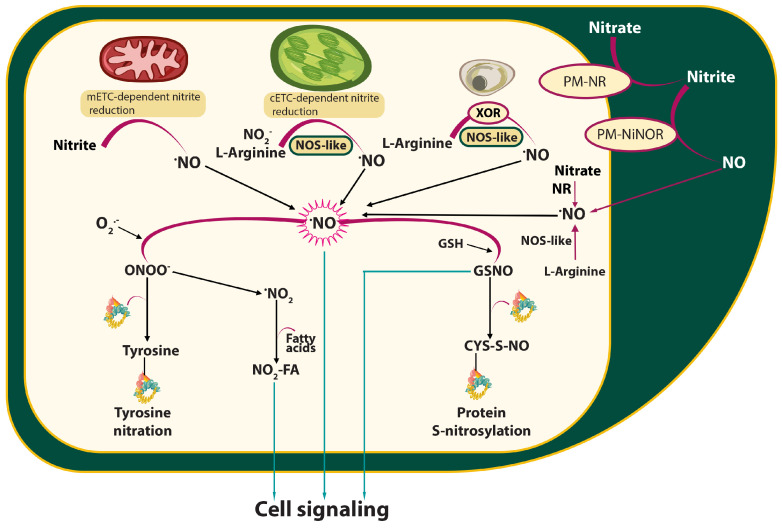
Simplified diagram showing the pathways of nitric oxide (NO) synthesis and transformation in plant cells. There are two main pathways for NO production in plants: the oxidative (arginine-dependent) and reductive (nitrite-dependent) pathways, involving different non-enzymatic or enzymatic mechanisms. NO synthesis in plant cells occurs primarily through nitrate reductase (NR)-mediated nitrite reduction. Plasma membrane nitrate reductase (PM-NR) is involved in the reduction of nitrate to nitrite, and then the enzyme nitrite: NO reductase (Ni-NOR/PM-NiNOR) can release NO into the apoplastic space. Reactive nitrogen species/NO interaction with free cysteine sulfhydryl groups leads to S-nitrosylation. On the other hand, peroxynitrite (ONOO^−^) mediates the nitration of tyrosine residues in proteins. Nitration and S-nitrosylation are necessary post-translational protein modifications mediated by NO, which are involved in plant signalling processes, similar to NO, GSNO, and nitro-fatty acids (NO_2_-FA). Abbreviations: cETC—chloroplastic electron transport chain; CYS—cysteine; CYS-S-NO—S-nitrosothiols; FA—fatty acid; GSH—glutathione; GSNO—S-nitrosoglutathione; mETC—mitochondrial electron transport chain; NO—nitric oxide; NiNOR—nitrite-NO reductase; NOS—nitric oxide synthase; PM—plasma membrane; PM-NR—plasma membrane nitrate reductase; XOR—xanthine oxidoreductase.

In plants, NO is produced through various pathways, which are categorised into reductive and oxidative processes [46]. The reductive pathways rely on nitrite as the primary substrate, and the generation of NO mediated by nitrate reductase (NR) was assumed to be the main enzymatic route in plants (Figure 1). However, under standard phototropic oxygenic conditions, the reduction of nitrite to NO constitutes only 1% of the activity of NR, which primarily catalyses the reduction of nitrate to nitrite [47]. Recently, it has been suggested that NR does not catalyse in vivo nitrate reduction to NO but instead provides electrons to the NO-forming nitrite reductase (NOFNiR), and this dual system NR-NOFNiR accounts for NO production and NO effects [48]. However, the functionality of the NR-NOFNiR system in higher plants remains unknown [45]. Other reductive NO-forming pathways include the plasma membrane-bound NR, NiNOR, which produces NO in the apoplast and mitochondrial electron transport chain (mETC)-dependent nitrite reduction to NO, as well as molybdenum cofactor-containing enzymes, such as xanthine oxidoreductase. To date, nitrite reduction is the best-evidenced NO synthesis pathway in plants [44]. In addition to the reductive pathways, oxidative NO production via an arginine-dependent route, similar to the nitric oxide synthase (NOS) activity found in animals, has been proposed in plants. While NOS-like activity has been reported in chloroplasts and peroxisomes in many plants, the corresponding enzymes have yet to be identified [44,49]. Additionally, molecules like S-nitrosoglutathione (GSNO) and nitro-fatty acids can directly release NO, serving as another endogenous source of NO in plants [49]. Recent studies on NO synthesis indicate that NO can also be produced in plant peroxisomes (similarly to animal cells) by the enzyme sulphite oxidase (SOX). The data indicate that peroxisomal SOX can produce NO from nitrite and NADH, and its activity is enhanced in the presence of H_2_S but is downregulated by H_2_O_2_ [50]. It is now widely accepted that multiple NO-producing pathways in plants can operate simultaneously, varying in extent depending on N nutrition and environmental conditions (Figure 1).

In the context of plant–pathogen interactions, NR was identified as a significant source of NO in *Arabidopsis thaliana* infected with *Pseudomonas syringae* and *Sclerotinia sclerotiorum* [51,52]. Furthermore, the HR response against an avirulent strain of *P. syringae* pv. *maculicola* was hindered in the *Arabidopsis* double mutant *nia1nia2*, which lacks the two structural genes for the NR enzyme. It was suggested that the mutation in NR influenced HR because the NR-deficient plants do not possess adequate L-arginine and NO_2_^−^, which are endogenous substrates for NO synthesis [53]. The production of NO during pathogenesis is also linked to arginine-dependent NOS-like activity. In *Arabidopsis*, the perception of a potential lipopolysaccharide PAMP from *Erwinia carotovora* activated AtNOS1 (nitric oxide synthase 1) and induced defence genes associated with both local and systemic responses. AtNOS1 mutants showed increased susceptibility to *P. syringae* pv. *tomato*, suggesting that NO generated by AtNOS1 is important for basal resistance in *Arabidopsis* [54]. Although AtNOS1, initially identified as a distinct plant NOS with no sequence similarity to enzymes in animals, was further characterised as a functional small GTPase and renamed AtNOA1 (nitric oxide associated 1), the AtNOA1 mutant is still a tool to study the NOS-like activity in plants [55,56]. In *Nicotiana benthamiana*–*Phytophthora infestans* interaction, NbNOA1, an AtNOA1 homolog, is involved in the production of NO and elicitor-induced resistance [57].

Much like ROS, time- and site-specific RNS production in plants is essential for pathogen recognition, activation of the defence response, and the outcome of the plant-pathogen interaction. In plants infected with leaf pathogens, local NO and ROS bursts occur in the epidermal cells during the early phase of pathogenesis. Both the plant and the pathogen contribute to the production of RONS (for review see [58] and references therein). These early RONS bursts are essential components of the plant immune response, which provides protection against biotrophic and hemibiotrophic pathogens both locally and systemically. Enhanced NO generation has been observed during ETI and HR, as well as PTI induced by different PAMP, such as cryptogein and oligogalacturonides [59,60]. At the local level, the successful defence reaction leads to HR and limits further biotrophic pathogen growth. In plant interactions with necrotrophic pathogens, however, HR-related ROS generation and oxidative stress can facilitate plant tissue colonisation and disease progression [61]. Therefore, the ability of NO to modulate the activity of ROS-generating NADPH oxidase D (RBOHD) through S-nitrosylation of Cys890 can serve as an essential controlling mechanism during HR, which can facilitate plant infection by necrotrophic pathogens [62,63]. In tomato interaction with *Botrytis cinerea*, a model necrotrophic fungal pathogen, the susceptible plant cultivar generated more ONOO^−^ and O_2_^−^ at the infection site and NO in chloroplasts, while generating less H_2_O_2_ and SNO compared to the resistant one. Furthermore, in tomato plants protected from this pathogen by *Trichoderma*, there was a rearrangement in the production, turnover, and location of the cooperating ROS and RNS, which coincided with enhanced defence responses [5]. Besides the local response, NO and ROS signalling are also involved in establishing systemic defence responses, which constitute systemic acquired resistance (SAR) [64,65]. NO/RNS are present in phloem and xylem tissues, and they mediate whole-plant signalling during SAR established in plant interactions with biotrophic and hemibiotrophic pathogens (for review see [66] and references therein). Under biotic stress, NO has also been suggested to mediate communication between plants, as infection results in enhanced NO emission [67].

In plant pathogens, both reductive and oxidative pathways of NO production, through nitrate/nitrite reductase and L-arginine-dependent mechanisms, have been identified, similar to those in plants [58]. In fungi and fungi-like oomycetes, NO regulates growth and development, including spore germination, mycelial growth, and active penetration of the plant tissue. Moreover, overproduction of NO by pathogens such as *B. cinerea* can promote pathogen virulence [68]. For biotrophic pathogens, maintaining a balance between NO and ROS levels produced in response to the invading pathogen appears to be crucial for successful invasion without triggering HR, which limits their propagation in plants. In necrotrophic pathogens, the overproduction of NO by the pathogen promotes necrotic cell death, facilitating the colonisation of host plants [58,68,69]. An interesting example is the role of NO in the interaction between *Ralstonia solanacearum*, the causative agent of bacterial wilt disease, and tomato plants. The pathogen survives in the hypoxic xylem by utilising denitrifying respiration, producing NO. To manage the toxic effects of NO and counteract plant-produced RNS, *R. solanacearum* uses protective proteins, including NorA, NorB, and HmpX. Bacterial NO can also signal plant defence responses. By maintaining low intracellular NO levels, the pathogen protects itself from toxicity and evades detection by the plant’s immune system. Inefficient detoxification of NO hampers its fitness and virulence [70]. Similarly, *P. infestans*, a representative of the oomycetes, possesses effective mechanisms for RNS elimination, including nitric oxide dioxygenase (Pi-NOD1), GSNOR, and peroxiredoxin (PRX2). This RNS detoxification system enables the pathogen to adapt to the host–plant microenvironment enriched in NO and may be implicated in its virulence [71].

Our understanding of nitric oxide (NO) catabolism in plants remains limited, much like our knowledge of its biosynthesis (Figure 1). NO reacts rapidly with superoxide anion radical (O_2_^−^), producing peroxynitrite (ONOO^−^). The availability of the precursors limits this reaction; therefore, in plants, ONOO^−^ is likely to be formed continuously in chloroplasts and peroxisomes as well as in response to pathogen infection, which induces the production of ROS and NO [72,73]. Peroxynitrite plays multifaceted roles in plants, acting as both a potential source of oxidative damage and a signalling molecule. At high levels, it induces lipid peroxidation and oxidative damage to proteins and DNA. During the induction of defence responses against pathogens, it has emerged as a potential signalling molecule, and its biological activity is mediated through the posttranslational modification of target proteins by tyrosine nitration [72]. Some of these proteins are involved in ROS metabolism, indicating a close relationship between ROS and RNS [73]. Similarly, ONOO^−^ mediates NO signalling during HR, a defence response against avirulent pathogens that involves the rapid and simultaneous generation of O_2_^−^ and NO [74]. The nitrated biotargets can affect signalling processes during plant–pathogen interactions, leading to adjustments in metabolism, including photosynthesis-related processes [75].

NO can quickly react with glutathione to form S-nitrosoglutathione (GSNO), a mobile reservoir of NO (Figure 1). The intracellular level of GSNO and the GSNO-mediated defence responses are controlled by GSNOR, which catalyses the reduction of GSNO to oxidised glutathione (GSSG) and NH_3_. In *Arabidopsis*, the depletion of *AtGSNOR* transcripts leads to disease resistance [76]. Inhibition of GSNOR activity via S-nitrosylation of the GSNOR protein has contributed to the enhanced disease resistance in post-harvest peach fruit to *Monilinia fructicola* [77]. In contrast, in tomato (*Solanum lycopersicum*), depletion of *SlGSNOR* function results in decreased salicylic acid biosynthesis and signalling, leading to compromised basal resistance to *P. syringae* [78]. Similarly, AtGNOR has been shown to positively regulate the SA signalling network [79]. Although the function of GSNOR in the molecular mechanism of plant immunity still requires elucidation, it has become evident that it is essential for multiple modes of plant disease resistance, and the levels of nitrosothiols play pivotal roles in the interactions of plants with pathogens [80,81].

GSNOR induces alterations in the intracellular redox status by regulating the levels of GSNO and other nitrosothiols, as well as the S-nitrosylation of defence-related proteins. S-nitrosylation can modify the structure, activity, and function of target proteins, which subsequently affects signal transduction pathways involved in various cellular processes, including plant defence responses. Recently, comprehensive reviews on this topic have been published [80,82].

During pathogenesis, RNS are produced and utilised in signalling pathways by both interacting partners, namely plants and pathogens. The role of NO in plant immune responses was first reported in potato tubers, where it was demonstrated to induce the accumulation of potato phytoalexin rishitin [83]. Since then, many specific functions of NO and RNS in pathogenesis have been uncovered. Most studies have used the model plant *Arabidopsis* to investigate its interaction with bacteria, leaving a significant gap in information regarding crop plants.

NO can mediate plant defence responses by controlling gene transcription. In *Arabidopsis*, transcription analysis revealed that NO-modulated genes are mostly regulatory proteins and transcription factors, such as WRKY, which are up-regulated in response to bacterial and fungal plant pathogens [84]. Results by Azzawi et al. [85] suggest that the NO-responsive gene, AtNIGR1 (*Arabidopsis thaliana* Negative Immune and Growth Regulator 1) involved in plant growth and immunity, is a negative regulator of basal defence and SAR, in *Arabidopsis* response to bacterial pathogens *P. syringae* pv. *tomato*.

## 3. The Role of NO in Regulating Stomata Movement

Stomata play a crucial role in gas exchange, influencing various metabolic processes, including photosynthesis, which depends on the availability of CO_2_, as well as respiration and transpiration. Stomata movement provides a short-term control of photosynthesis and transpiration [86,87]. Plant stomata are also important mediators of interactions between plants and pathogens. Pathogens such as *P. syringae* and *Xanthomonas campestris* have evolved distinct strategies, including the production of toxins and effectors, to induce stomatal opening and facilitate invasion [88,89]. Stomatal closure, often accompanied by water-soaking symptoms, is a typical plant response to leaf pathogen attack. It is initiated by sensing PAMP (e.g., flagellin 22) or plant hormones (e.g., abscisic acid, ABA) by the pattern-recognition receptors on the guard cells [88]. Its biological significance lies in restricting pathogen entry as the first line of defence (PAMP-triggered stomata defence), but the events initiated during closure contribute to a long-term response against microbes [90]. For example, resistant plants close their stomata to inhibit pathogen entry, but they reopen them to promote leaf transpiration and limit the post-invasive multiplication of the pathogen, establishing a second layer of stomatal defence termed water immunity [91].

NO promotes stomatal closure, and this is suggested to depend on H_2_O_2_. When the pattern recognition receptors detect a pathogen, guard cells initiate the production of ROS, NO, and release Ca^2+^ from Ca^2+^ stores into the cytoplasm [88,92]. These secondary messengers cause ion efflux from the guard cells, leading to the closure of stomata. The signalling pathway involves several factors like: OPEN STOMATA 1 (OST1)/SNF1-RELATED PROTEIN KINASE 2.6 (SnRK2.6), plasma membrane-resident ion channel SLOW ANION CHANNEL ASSOCIATED 1 (SLAC1), and S-TYPE ANION CHANNEL SLAH3 as well as hormone ABA, (for review, see [93]). NO negatively regulates ABA signalling in guard cells by S-nitrosylation of SnRK2.6 [94].

Pathogen elicitors induce ROS accumulation in guard cells, activating the ROS-generating AtrbohD and AtrbohF NADPH oxidases. ROS act upstream of NO and are necessary for NO production in guard cells, which is suggested to depend on NR and NOS-like activity [95,96,97]. A feedback regulatory effect of NO on ROS production, mediated by protein S-nitrosylation, has been suggested. NADPH oxidase’s N-nitrosylation inhibits its ability to produce ROS, while the H_2_O_2_-scavenging activity of ascorbate peroxidase is positively regulated by S-nitrosylation [63,98,99]. Moreover, pathogen-derived PAMP and effectors interfere with ABA-mediated signalling to manipulate the stomata movement and counteract stomata immunity [100]. Therefore, the reciprocal interactions of NO with ROS and ABA are of great physiological significance in the stomata-mediated plant resistance to pathogens.

Aquaporins, intrinsic membrane proteins (PIP) that facilitate the transport of H_2_O as well as H_2_O_2_, CO_2_, and NO, are considered important regulators of stomata movement and are involved in plant responses to pathogens. In *Arabidopsis*, the bacterial pathogen PAMP flg22 can activate OST1 and BAK1 kinases to phosphorylate Ser121 of aquaporin AtPIP2;1 and close the stomata to restrict the pathogen entry [101]. Moreover, the PIP channels facilitate the translocation of pathogen-induced H_2_O_2_ from the apoplast to the cytoplasm, which may intensify the H_2_O_2_-mediated defence responses [102]. Aquaporin expression and functioning in plants are likely to be regulated by NO-dependent S-nitrosylation [103].

In addition to being an important component of early defence against pathogens, stomatal closure also has a long-term impact on plant-pathogen interactions. It restricts the entry of CO_2_, limits photosynthesis, decreases leaf sugar levels, and reduces water transpiration, thereby raising leaf temperature, which in turn increases photorespiration and may lead to mineral deficiencies in leaves [90]. Reduced transpiration creates mineral deficiency in leaves, and the leaf N-status modulates NO content and the spread and multiplication of the leaf pathogens [104]. The photorespiratory metabolism may assist in resisting pathogens by producing stress-signalling molecules, such as H_2_O_2_ and NO. Additionally, its metabolites, namely glycolate, glyoxalate, and glycine, mediate plant defence against pathogens [34]. Glyoxalate and glycolate are antimicrobial compounds, while glycine serves as a precursor to glutathione, which interacts with NO to produce GSNO. This endogenous NO reserve contributes to the intracellular regulation of stress defence [73,77,105].

The direct impact of stomatal closure on leaf sugar levels has been questioned, particularly in plants infected with *P. syringae*, which is the most extensively studied bacterial pathogen. In these pathosystems, the net rate of CO_2_ assimilation was restricted more by photochemical and biochemical factors than by the closure of stomata [18].

Decreased CO_2_ assimilation caused by NO-mediated stomatal closure can lead to lower leaf sugar content. This could restrict pathogen proliferation in the infected tissues, as sugars, particularly sucrose, are the primary carbon sources that pathogens absorb from host cells [106,107]. On the other hand, lower sugar content can impair resistance response, as sugar plays a key role in coordinating plant defence signalling [108]. In *Arabidopsis*, the application of exogenous glucose and fructose enhanced resistance to *P. syringae* pv. *tomato*, significantly increasing the endogenous NO level. This indicates that sugars are involved in the plant’s innate immunity against this bacterial pathogen through a NO-dependent pathway [109].

Despite some remaining questions, the NO-mediated stomatal closure induced by pathogens seems to both directly assist in limiting the multiplication, spread, and growth of invading microbes and to interfere with the plant’s immune signalling.

## 4. The Role of NO in the Regulation of Photosynthesis

Photosynthesis is sensitive to pathogen-induced biotic stress. Photosynthesis and plant defence against pathogens are interconnected through a complex network. Photosynthesis affects signalling and provides energy, reducing equivalents, and carbon skeletons for defence, while the immune response also impacts photosynthesis [18,110,111]. Although the effects of pathogens on photosynthesis vary depending on the pathogen’s lifestyle and the infection phase, many pathosystems show a decline in photosynthesis at infection sites. These changes occur earlier and are more pronounced in resistant interactions [18,107,112]. Therefore, changes in photosynthesis during the early stages of infection have been suggested as an indicator of resistance level [19]. In remote plant tissues, photosynthesis remains unchanged or is upregulated [107,113,114].

During pathogen attack, the formation of NO occurring in chloroplasts contributes to the specificity of the immune reaction [115]. In tobacco epidermal cells, chloroplasts were the first organelles to accumulate NO within minutes after treatment with cryptogein, a protein elicitor derived from *P. cryptogea* [116]. Chloroplasts are also the primary source of ROS and interact with other organelles, such as peroxisomes and mitochondria, which produce RONS, to establish a RONS signalling network that triggers a significant subset of PTI and ETI responses, e.g., HR-related programmed cell death and induction of defence genes (for recent reviews see [31,42,117,118]. The effects of NO on the plant photosynthetic system have been extensively studied, resulting in the identification of numerous target sites of NO action in chloroplasts and the points of interaction between NO and ROS [40,119]. When reacting together, NO and ROS can produce other signalling molecules, such as ONOO^−^, which is an important protein-nitrating agent [72,74]. RNS and ROS can also compete for downstream molecular targets, such as thiols and proteins, thereby modulating the infection-induced stress signature [40,80,105]. Moreover, ROS/NO and SA act in parallel to confer SAR [120]. In the leaves of citrus plants, proteomic analysis has shown that the largest group of proteins regulated by H_2_O_2_ and NO is associated with photosynthesis, particularly the Calvin–Benson cycle [121,122]. These findings suggest that under conditions of nitro-oxidative stress, which also occurs in infected plants, photosynthetic proteins are the primary targets for H_2_O_2_ and NO interacting in stress signalling.

Light is a significant determinant of plants’ photosynthetic rate and sugar synthesis, while also playing a central role in shaping their local and systemic defence responses against pathogens [123,124]. Pathogen infection induces stomatal closure and inhibits photosynthesis, resulting in episodes of absorbed excess energy. This, along with the quality of the light spectrum, is sensed by chloroplasts [125]. To optimise photosynthetic efficiency and protection against stress, plants maintain a balance between light absorption, energy dissipation and redox regulations. The chloroplast-based photoprotective mechanisms include cyclic electron flow, the non-photochemical quenching (NPQ) and plastid terminal oxidase [126]. Chloroplasts sense light intensity through changes in plastoquinone’s redox state in the electron transport chain, and signals emerging from the plastoquinone pool indirectly orchestrate plant response to pathogens [127]. NO production in plants is also regulated by light. In *Arabidopsis* plants, exposure to increasing light intensities enhanced NO emission as well as nitrite and S-nitrosothiols accumulation [128]. This is linked to the circadian regulation of NR activity, which is post-translationally activated in the presence of light and inactivated in the dark. Consistent with lower activity of NR and the concentration of NO_2_^−^, spinach leaves emitted less NO in the dark than in the light. The regulation of NR activity by light can be vital for adjusting NO production to the light conditions [129].

Light not only influences the NR-dependent NO generation, but also NO degradation. The GSNOR-mediated degradation of GSNO, which serves as the primary mobile reservoir of NO in plants, is also enhanced by light. GSNOR, required to fine-tune the S-nitrosothiols levels, plays an important role in the light-dependent regulation of histone acetylation and expression of photosynthesis genes [128]. Consistently, the *gsnor Arabidopsis* mutants have altered photosynthetic properties, such as increased quantum efficiency of photosystem II (PSII) and photochemical quenching, and decreased nonphotochemical quenching [122].

NO may exert pleiotropic effects on the photosynthetic metabolism. However, the exact mechanism by which NO affects photosynthesis remains unknown. The results on the effects of NO on photosynthesis, regarding its toxicity or benefits, are controversial. This is likely partly due to the different NO donors that were used, as studies investigating the roles and effects of NO in photosynthesis predominantly utilise exogenous application of NO [130,131]. In pea leaves, studies with S-nitrosoglutathione, which effects are inferred exclusively by NO, indicated an inhibitory effect of NO on photosynthetic electron transport, modulation of NPQ, and up-regulation of cyclic electron transport, possibly to balance the damage to PSII [132,133]. In pea mesophyll protoplasts treated with sodium nitroprusside (NO donor), NO decreased the rate of photosynthesis, and the PSI activity was more sensitive to NO than PSII [134]. In maize and sorghum, sodium nitroprusside was suggested to enhance photosynthetic performance by increasing the number of open PSII reaction centres and their efficiency [135]. These conflicting results arise from the differing chemical properties of NO donors and varying experimental conditions.

The available information on the effect of NO on photosynthesis under stress stems from studies on plants subjected to abiotic stress factors, indicating that applying NO can help mitigate the stress-induced decline in photosynthesis [136]. For example, in Chinese cabbage exposed to low light, exogenously applied NO enhanced the net photosynthetic rate (P_N_) and the relative electron transport rates (rETR) [137]. In mustard plants (*Brassica juncea*) exposed to salinity, NO treatment enhanced the photosynthetic capacity as shown by increased maximal PSII photochemical efficiency (Fv/Fm), P_N_, stomatal conductance (g_s_) and intracellular CO_2_ concentration (C_i_) [138]. Similarly, in maize under salt stress, NO enhanced the PSII performance and overall function of the photosynthetic apparatus by mitigating the salt-induced changes on both the donor and acceptor sides of PSII [139]. In pea (*Pisum sativum*), NO application alleviated the drought-induced oxidative damage by enhancing P_N_, g_s_, and the antioxidant defence. It also improved the endogenous NO content [140]. Interestingly, priming with NO-releasing nanoparticles such as chitosan-encapsulated NO donors increased the endogenous NO level in plants. This NO-associated priming has been hypothesised to improve plant photosynthesis, growth and biomass production under abiotic and biotic stress [141]. It remains to be elucidated whether the same NO-mediated mechanisms can operate in infected plants to support their defence mechanisms, while minimising the adverse effects of pathogens on growth and productivity.

NO was reported to be a reversible inhibitor of photosynthetic ATP synthesis. In spinach chloroplasts, NO inhibited the linear electron transport rate, light-induced ΔpH across the thylakoid membranes and photophosphorylation [131]. The authors suggested that under stress, when chloroplast electron transport is inhibited or nitrite reduction in chloroplasts is non-operational, the NR-produced NO downregulates photosynthesis, acting as a messenger of dysfunctional nitrogen assimilation.

Given that NO from the RONS burst is an important signal generated upon pathogen infection in plants, the inhibitory effects on photosynthesis observed in plants treated with NO donors suggest that NO plays a role in suppressing photosynthesis shortly after pathogen infection by inducing stomatal closure. This plant defence strategy may limit resources available to biotrophic pathogens, reprogramme plant metabolism for defence rather than growth, and generate defence signals that activate local and systemic responses. In accordance, recent studies have shown that a plant’s ability to rapidly and continuously increase NO production is part of the molecular mechanism of its resistance to biotrophic pathogens [142].

Besides perturbations in the photochemical processes, carbon-assimilation reactions are also influenced by NO, for example, by affecting the level/activity of several critical photosynthetic proteins. Ribulose-1,5-bisphosphate carboxylase/oxygenase (Rubisco) activase protein and Rubisco subunit binding protein β-subunit were down-regulated in NO-treated mung bean leaves [143]. All key enzymes in the Calvin cycle have been found to be S-nitrosylated; however, the biological significance of this modification still needs to be clarified [122]. Rubisco and glyceraldehyde-3-phosphate dehydrogenase (GAPDH) are also regulated by this post-translational modification, with S-nitrosylation inhibiting their activities [144,145]. Interestingly, some proteins are targets of both NO and H_2_O_2_. GAPDH, which is the leading consumer of photosynthetic NADPH and, as such, is tightly regulated, is targeted by H_2_O_2_ and NO via S-nitrosylation [146,147]. It cannot be excluded that this S-nitrosylation-based regulation reduces Calvin cycle flux in order to transiently redistribute sugars, energy (ATP), and reducing equivalents (NAPDH) from the primary metabolism to pathogen defence.

In tomato plants infected with *P. infestans*, the chloroplast ATP synthase CF1 alpha subunit was identified as a S-nitrosylation target [122,148]. Under pathogen-induced oxidative stress, the increased S-nitrosylation of ATP synthase has been suggested to serve as a redox-mediated modulation of the cellular ATP levels [148].

The collapse of photosynthetic activity leads to a metabolic transition from source to sink in infected tissues, facilitating the acquisition of nutrients, particularly sugars, and the pathogen’s spread [149]. Generally, infection by biotrophic pathogens creates a new sink which competes with existing sinks [150]. The cell-wall invertase hydrolysing apoplastic sucrose is the key enzyme supplying carbohydrates to sink tissues, and its increased activity has been reported in different pathosystems [112,151]. Interestingly, invertase of pathogen origin can also contribute to the increased invertase activity in the infected plant tissues. For example, the rust fungus (*Puccinia striiformis* f. sp. tritici), an obligatory biotrophic pathogen, secretes a specific invertase during infection of wheat to ensure effective sugar absorption and support its growth and development [106].

High apoplastic sugar content can be beneficial for plants as sugars serve as signalling molecules that activate defence responses, including pathogenesis-related genes, as reviewed by [152]. Recent studies, however, have shown that during pathogenesis, invertase may act as a resistance and susceptibility factor depending on the plant–pathogen interaction [108,153].

NO can modulate invertase activity in plants, and its exogenous application regulates the activities of invertases in wheat, cucumber, and lemongrass [154,155,156]. Although the results with NO donors should be interpreted with caution, it is reasonable to speculate that NO generated in the infected plants may also modulate invertase activity, thereby influencing carbohydrate metabolism and the overall response to infection. This regulatory role of NO is supported by findings indicating that NO significantly inhibits monosaccharide catabolism by modulating sugar metabolic enzymes through S-nitrosylation. In *Arabidopsis*, these S-nitrosylation modifications lead to the inhibition of starch biosynthesis and the accumulation of hexoses, such as glucose and fructose [157].

**Figure 2:**
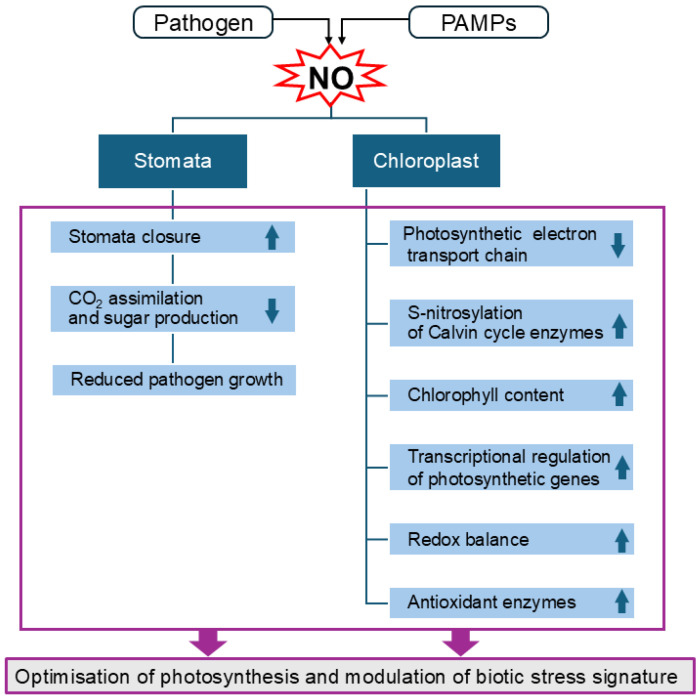
Nitric oxide action on stomata and its target sites in chloroplasts during plant–pathogen interactions. Plant cells overproduce NO upon pathogen/PAMPs sensing. NO from the nitrosative burst mediates stomatal closure to restrict microbial pathogen entry at the sites of infection, which is a part of the plant’s innate immunity response, termed stomatal immunity. Some bacterial pathogens manipulate stomatal aperture to promote pathogenicity. While stomatal closure is crucial for defence, it also restricts the uptake of CO_2_, resulting in decreased photosynthesis rates and lower production of assimilates. The reduced availability of sugars limits pathogen proliferation, since pathogens rely on sugars for their growth and infection. In chloroplasts, NO increases chlorophyll content and tightly controls photosynthetic activity. The NO target sites in the photosynthetic electron transport chain include the oxygen-evolving complex, ATP synthase, cyt b_6_f complex, PSI, and PSII. NO mediates post-transcriptional regulations of photosynthetic genes, including key enzymes of the Calvin cycle, with Rubisco and Rubisco activase being under dual NO-triggered regulations via S-nitrosylation and tyrosine nitration. NO production promotes the expression of antioxidant enzymes, e.g., APX and SOD, preventing cellular damage and balancing the redox equilibrium. These NO-mediated regulations not only restrict microbial pathogen entry through stomatal closure, but also optimise photosynthesis under biotic stress and modulate the chloroplast-derived stress signalling, shaping the local and systemic responses to pathogens. Abbreviations: APX—ascorbate peroxidase; PAMP—pathogen-associated molecular pattern; PSI—photosystem I; PSII—photosystem II; SOD—superoxide dismutase.

In general, NO, which is produced excessively following a pathogen attack, can play a dual role in infected plants (Figure 2). Initially, NO can reduce photosynthetic activity by causing stomatal closure. However, it also activates protective mechanisms that help mitigate nitro-oxidative damage and maintain the integrity of the photosynthetic system. Additionally, NO mediates defence responses by regulating signalling pathways and gene expression [126]. Nevertheless, the functional significance of these mechanisms in specific plant–pathogen interactions remains to be clarified.

## 5. Protein S-Nitrosylation in Regulating Plant Immune Responses

**Figure 3:**
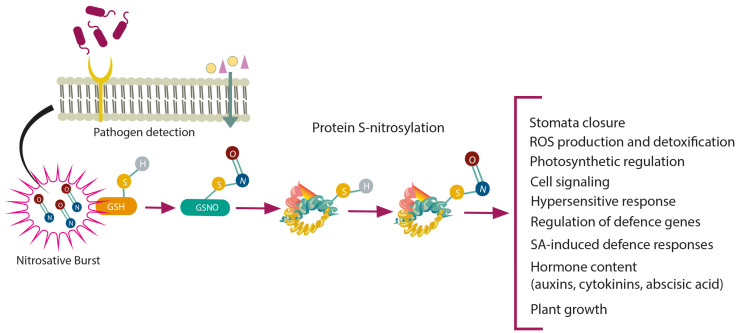
Multiple functions of S-nitrosylation in plants following pathogen infection. In response to infection, upon recognition of the pathogen, reactive nitrogen species, including nitric oxide (NO), are rapidly produced in plant cells, leading to nitrosative burst. The reaction of NO with glutathione (GSH) leads to the production of S-nitrosoglutathione (GSNO), which acts as a mobile reservoir of NO bioactivity. NO may bind covalently to specific reactive cysteine thiols of various proteins to form S-nitrosothiols (SNO), which are stored in the plant cell. Under biotic stress, S-nitrosylation controls the production and detoxification of reactive oxygen species (ROS). S-nitrosylated proteins are involved in regulating metabolism, growth, and developmental processes, as well as programmed cell death, and salicylic acid (SA) and other phytohormone signalling, which are integrated into the plant defence response. Abbreviations: ROS—reactive oxygen species; SA—salicylic acid.

In plants, the biological activity of NO is primarily mediated by protein S-nitrosylation, a redox-based post-translational modification that covalently attaches an NO group to the reactive Cys thiol (-SH) to form S-nitrosothiol (-SNO) (Figure 3). This reversible process, which can modify protein activity, stability, localisation, and protein–protein interactions, has emerged as a fundamental process for cellular signalling [81,158]. The level of S-nitrosylation is controlled indirectly by the action of GSNOR [159].

Recent technological advances have enabled complex studies on S-nitrosylation in plant tissues and have shown that it modifies a wide range of proteins/enzymes involved in metabolic reactions, but also in hormone signalling and stress perception, response to stress factors, as well as the antioxidant enzymes of the ascorbate–glutathione cycle and catalase [122,160,161]. Such regulation provides flexible responses to various stress factors, optimises hormonal balance and growth under different environmental conditions. Several examples of protein S-nitrosylation and its role in plant growth and development, as well as under abiotic stress conditions, have been provided in recent reviews [158,162,163]. Much less is known about the role of this modification after pathogen infection [65,80]. In addition, most studies are based on the experimental interaction model: *A. thaliana*–*P. syringae*. In this pathosystem, NO, which accumulates significantly during ETI, modifies proteins through S-nitrosylation. Most S-nitrosylation-modified proteins are involved in photosynthetic processes, including the PSII oxygen evolution complex and the large Rubisco subunit. This NO-mediated protein S-nitrosylation may therefore provide a link between ETI and altered photosynthetic function [146,164,165]. Moreover, plants can utilise S-nitrosylation to circumvent the suppression of ETI signalling mediated by pathogen effectors. In *Arabidopsis*, at the onset of HR, the *P. syringae* effector HopAI1 undergoes S-nitrosylation, which restores the signalling necessary for activating HR-associated cell death [166].

Another way NO modulates the plant immune response is by regulating SA-linked target proteins, such as NPR1. NPR1 is a critical component of both SAR and ETI signalling. Plant immunity requires redox-dependent conformational changes of NPR1 via S-nitrosylation and thioredoxins. The perception of a pathogen or activation by SA leads to changes in cytoplasmic redox state, which trigger the S-nitrosylation of the Cys156 residue in NPR1. This modification facilitates the formation of transient tetrameric NPR1 complexes. Thioredoxins (TRX-h3 and TRX-h5) reduce Cys156, causing the disassembly of the NPR1 oligomeric form. The released monomeric NPR1 then translocates into the nucleus, where it interacts with TGA transcription factors to induce changes in gene expression [167,168,169,170]. Moreover, NO accumulation during the nitrosative burst after infection promotes S-nitrosylation of the *Arabidopsis* SA-binding protein 3 (AtSABP3) at Cys280, which not only suppresses its binding to SA but also inhibits its carbonic anhydrase activity. As the carbonic anhydrase function of AtSABP3 is required for the expression of plant resistance against attempted pathogen infection, its activity inhibition could negatively regulate the immune response [171].

Selected examples of plant protein S-nitrosylation are presented in Table 1, with particular emphasis on plant–pathogen interactions. Further research and understanding of this process will likely enable the manipulation of S-nitrosylation in crop plants, thereby increasing resistance to stress factors and enhancing yield stability in agriculture.

Besides post-transcriptional modifications, such as S-nitrosylation and tyrosine nitration, the NO-sensing mechanism in plants also involves the ubiquitin–proteasome pathway [172]. NO is involved in the ubiquitin-mediated proteasomal degradation, such as the N-degron pathway, which plays a role in regulating the amplitude and timing of the immune response in the model *Arabidopsis*–*P. syringae* interaction [173].

**Table 1:** S-nitrosylated proteins/enzymes operating in various reactions in plant cells and their involvement in plant–pathogen interactions.

Nitrosylated Protein	Function in Plant Cells	Plant–Pathogen Interaction	S-Nitrosylation Effects	References
NPR1 (Non-Pathogenesis Related 1)	Non-expressor of Pathogen Related genes 1	*Arabidopsis thaliana*–*Pseudomonas syringae* pv. *maculicola*	S-nitrosylation of cysteine 156 in NPR1 facilitates oligomerization/retention in cytosol (thioredoxin-h 5, TRXh5-capacity to denitrosylate Cys156 of NPR1)	[169]
S-nitrosylation of the NADPH oxidase, AtRBOHD, at Cys 890	NADPH oxidases generate ROS after pathogen recognition	*A. thaliana*–*P. syringae* pv. *tomato*	AtRBOHD S-nitrosylation at Cys890 reduces ROS production during pathogen attacks, thus weakening the development of HR	[63]
SABP3-salicylic acid binding protein 3	Role during the establishment of plant disease resistance; possesses carbonic anhydrase activity	*A. thaliana–P. syringae*	AtSABP3 S-nitrosylated at Cys280 inhibition of SA-binding activities; negative regulation of SA-dependent defence response at the later stage of infection	[171]
SCE1-small ubiquitin-like modifier (SUMO)-conjugating enzyme 1	Inhibition of SCE1 enzyme activity facilitates SA-dependent immune responses	*A. thaliana*–*P. syringae* pv. *tomato*	S-nitrosylation of SCE1 at Cys139 stimulates *PR1* expression and enhances plant immunity	[174]
SRG1-zinc finger transcription factor (ZF-TF)	NO accumulation promotes SRG1 expression	*A. thaliana*–*P. syringae* pv. *tomato*	S-nitrosylation of SRG1 at Cys87 positively regulates plant growth and immunity	[175]
HopAI1, bacterial effector HopAI1	HopAI1 targets and suppresses mitogen-activated protein kinases (MAPK)	*A. thaliana*–*P. syringae* pv. *tomato*	S-nitrosylation of HopAI1 restores MAPK signalling and is required during the HR for activation of the HR-associated cell death	[166]
CDC48, chaperon-like protein	Chaperon-like protein Cdc48, cryptogein-induced immune response	*Nicotiana tabacum*–*Phytophthora* *cryptogea*	S-nitrosylation of NtCDC48 at Cys526 compromises immune responses in plant cells	[80,176]
COMT2– caffeic acid *O*-methyltransfe-rase 2	NO and JA enhanced COMT-mediated infection-induced melatonin biosynthesis; melatonin inhibited cell death by scavenging ROS	*Solanum lycopersicum*–*Botrytis cinerea*	S-nitrosylation of SlCOMT2 at Cys344, enhances COMT2 stability and prevents its degradation via the 26S proteasome	[177]
GDC glycine decarboxylase complex (EC 2.1.2.10)	Mitochondria-localised GDC is a key enzyme of photorespiration in C3 plants	*A. thaliana*–harpin proteins (bacterial elicitors of *Erwinia* and *Pseudomonas*)	S-nitrosylation and S-glutathionylation inhibit GDC activity	[178]
Pyruvate kinase (EC 2.7.1.40)	Cytoplasm-localised, glycolysis enzyme	*A. thaliana*–*P. syringae* pv. *tomato*	S-nitrosylation of pyruvate kinase probably regulates enzyme activity	[179]
PrxII E peroxiredoxin	Chloroplast-localized peroxiredoxin, efficiently removes H_2_O_2_	*A. thaliana*–*P. syringae* pv. *tomato*	S-nitrosylation of PrxII E at Cys121 leads to the inhibition of both the peroxidase and ONOO^−^ reductase activities	[180]
Fructose-1,6-bisphosphatase (FBPase, EC 3.1.3.11)	Calvin–Benson cycle enzyme converting Fru-1,6-BP to Fru-6-P and Pi	*Pisum sativum*	S-nitrosylation at Cys153 of cFBP1 (FBPase isoform) leads to the formation of a regulatory disulfide bridge	[181]
Ribulose 1,5-bisphosphate carboxylase/oxygenase (Rubisco, EC 4.1.1.39)	Chloroplast-stroma localized key enzyme of the Calvin–Benson cycle	*A. thaliana*–*P. syringae* pv. *tomato*	S-nitrosylation of large/small Rubisco subunits likely regulates its activity and turnover	[146,164,179,182]
Rubisco activase	Calvin–Benson cycle enzyme activation	*A. thaliana*–*P. syringae* pv. *tomato*	S-nitrosylation of Rubisco activase detected	[182]
Rubisco large subunit-binding protein (subunit alpha)	Chloroplastic chaperone	*A. thaliana*–*P. syringae* pv. *tomato*	S-nitrosylation influences chloroplast organization-protein folding/chaperone	[179]
PSBP-1 PSBO2	Photosystem II subunits	*A. thaliana*–*P. syringae* pv. *tomato*	S-nitrosylation of PS II subunits detected	[182]
23 kDa subunit of oxygen evolving system of PSII	Photosystem II protein	*A. thaliana*–*P. syringae* pv. *tomato*	S-nitrosylation of 23 kDa subunit of PSII detected	[179]
Glyceraldehyde-3-phosphate dehydrogenase (GAPDH, EC 1.2.1.13)	Calvin–Benson cycle enzyme	*A. thaliana*–*P. syringae* pv. *tomato*	S-nitrosylation of B subunit of GAPDH inhibits its activity	[179]
CPCK2-α subunit of casein kinase II (CK2, Ser/Thr kinase)	Chloroplast-localised protein kinase CK2 subunit, CPCK2 regulates various pathways in plants	*A. thalina*–*Golovinomyces cichoracearum* *A. thaliana*–*P. syringae* pv. *tomato*	CPCK2 negatively regulates plant immunity by promoting S-nitrosylation of SABP3; *cpck2* mutants accumulate SA and show resistance against the fungal pathogen powdery mildew	[183]
MPK6 protein kinase (a part of MAPK cascade)	MPK6 controls stomatal development by phosphorylating transcription factor SPCH	*A. thaliana* mutants treated with ABA	S-nitrosylation of MPK6 at Cys201 promotes stomatal development and controls stress responses	[184]

## 6. Conclusions and Future Perspectives

**Figure 4:**
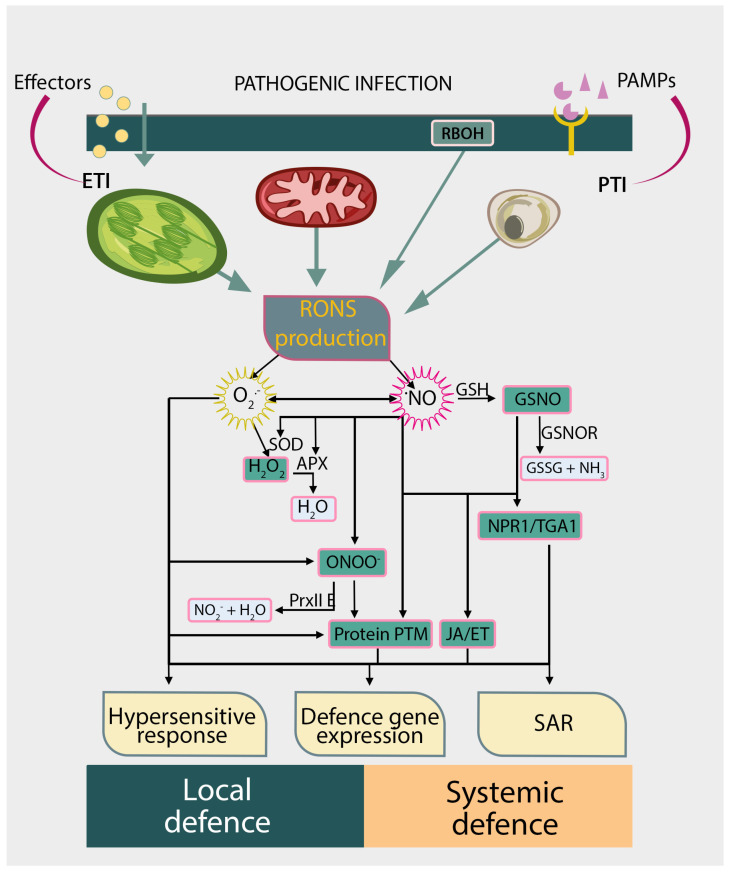
Schematic presentation of nitric oxide (NO) action mechanisms during plant–pathogen interactions. After infection, plants exhibit defensive reactions, classified as local or systemic defence reactions. Plant cells perceive pathogen-expressed PAMPs or effectors (via various receptors) and initiate pattern/effector-triggered immunity (PTI or ETI). After the perception of PAMP or effectors, reactive oxygen and nitrogen species (RONS) production increases, including ROS synthesis by plasma membrane NADPH-oxidase homolog, RBOHD. The reaction of NO with glutathione (GSH) leads to the production of S-nitrosoglutathione (GSNO), controlled by S-nitrosoglutathione reductase (GSNOR). RONS production can lead to a hypersensitive response (HR) at the site of infection. NPR1 protein is considered to be a crucial regulator of salicylic acid-mediated gene expression in systemic acquired resistance (SAR). The complex interactions of NO with other signalling pathways, including reactive oxygen species-mediated redox regulation, hormone signalling (such as stress hormones JA or ET), and post-translational protein modifications, lead to changes in defence gene expression and modulate the local and systemic defence responses. Abbreviations: APX—ascorbate peroxidase; ET—ethylene; ETI—effector-triggered immunity; GSH—glutathione; GSNO—S-nitrosoglutathione; GSNOR—S-nitrosoglutathione reductase; GSSG—glutathione disulfide; H_2_O_2_—hydrogen peroxide; JA—jasmonic acid; NO—nitric oxide; ONOO^−^—peroxynitrite; NH_3_—ammonium; NPR1—nonexpresser of pathogenesis-related genes1, transcription factor; NR—nitrate reductase; PAMPs—pathogen-associated molecular patterns; PrxII—peroxiredoxinII; PTI—pattern-triggered immunity; PTM—post-translational modifications; RBOH—respiratory burst oxidase homolog protein; RONS—reactive oxygen and nitrogen species; SAR—systemic acquired resistance; SOD—superoxide dismutase; TGA—family of basic leucine zipper transcription factors (binding TGACG motif).

In recent decades, significant progress has been made in understanding how NO is produced and degraded in plant tissues. More and more is known about the role of NO/RNS in plant stress responses. Undoubtedly, NO is one of the key molecules in stress signalling, which interacts with numerous signalling pathways and affects changes in gene expression and post-translational modifications of proteins. These NO-mediated processes modulate the local and systemic defence against pathogens (Figure 4). The mechanism of NO action is best understood in *A. thaliana*, but more recent studies indicate differences between this model plant and crop species important for agriculture and food security.

Numerous studies have explored the role of NO in plants under abiotic stress and its contribution to enhancing stress tolerance [9,135,163]. NO is involved in regulating redox homeostasis, stomata conductance, and gene expression, which are essential for maintaining photosynthetic efficiency. As NO regulates photosynthesis and stomata movements, which are the determinants of plant carbon gain and productivity, NO is considered a valuable tool for improving both the yield and quality of crops grown in unfavourable environmental conditions. However, there is less information on the role and mechanisms of NO action in biotic stress, which leads to significant yield losses in crops. There are notable gaps regarding the interactions between RONS and photosynthetic metabolism in plants infected with pathogens. Photosynthesis is a vital process that drives plant growth and biomass production, while also enhancing the immune system and aiding plant defence against pathogens. Given the complex roles of NO in plants, NO signalling appears to be a promising target for optimising growth and defence processes under pathogen-induced biotic stress [43,134]. Nevertheless, further comprehensive studies are necessary to understand the molecular mechanisms of NO action and the role of NO/RNS produced by plant cells and pathogens in effective plant defence, and to propose strategies for enhancing crops’ resistance to pathogens, which is vital for agriculture and future crop productivity.

## Data Availability

Not applicable.
